# Intestinal absorption of sphingosine: new insights on generated ceramide species using stable isotope tracing in vitro

**DOI:** 10.1016/j.jlr.2024.100557

**Published:** 2024-05-07

**Authors:** Catherine Calzada, David Cheillan, Nina Ritsch, Cécile Vors, Annie Durand, Sandra Pesenti, Magali Pettazzoni, Emmanuelle Meugnier, Marie-Caroline Michalski, Armelle Penhoat

**Affiliations:** 1 CarMeN Laboratory, Inserm U1060, INRAE UMR1397, Univ-Lyon, Université Claude Bernard Lyon-1, Pierre Bénite, France; 2 Service de Biochimie et de Biologie Moléculaire, Centre de Biologie et de Pathologie Est, Hospices Civils de Lyon, Bron, France

**Keywords:** chylomicrons, dietary fat, enterocytes, lipidomics, mass spectrometry, milk, polar lipids, sphingolipids, sphingomyelin, triglycerides

## Abstract

Dietary sphingomyelin (SM) has been reported to favorably modulate postprandial lipemia. Mechanisms underlying these beneficial effects on cardiovascular risk markers are not fully elucidated. Rodent studies showed that tritiated SM was hydrolyzed in the intestinal lumen into ceramides (Cer) and further to sphingosine (SPH) and fatty acids (FA) that were absorbed by the intestine. Our objective was to investigate the uptake and metabolism of SPH and/or tricosanoic acid (C23:0), the main FA of milk SM, as well as lipid secretion in Caco-2/TC7 cells cultured on semipermeable inserts. Mixed micelles (MM) consisting of different digested lipids and taurocholate were prepared without or with SPH, SPH and C23:0 (SPH+C23:0), or C23:0. Triglycerides (TG) were quantified in the basolateral medium, and sphingolipids were analyzed by tandem mass spectrometry. TG secretion increased 11-fold in all MM-incubated cells compared with lipid-free medium. Apical supply of SPH-enriched MM led to increased concentrations of total Cer in cells, and coaddition of C23:0 in SPH-enriched MM led to a preferential increase of C23:0 Cer and C23:0 SM. Complementary experiments using deuterated SPH demonstrated that SPH-d9 was partly converted to sphingosine-1-phosphate-d9, Cer-d9, and SM-d9 within cells incubated with SPH-enriched MM. A few Cer-d9 (2% of added SPH-d9) was recovered in the basolateral medium of (MM+SPH)-incubated cells, especially C23:0 Cer-d9 in (MM+SPH+C23:0)-enriched cells. In conclusion, present results indicate that MM enriched with (SPH+C23:0), such as found in postprandial micelles formed after milk SM ingestion, directly impacts sphingolipid endogenous metabolism in enterocytes, resulting in the secretion of TG-rich particles enriched with C23:0 Cer.

## Graphical abstract

**Figure:**
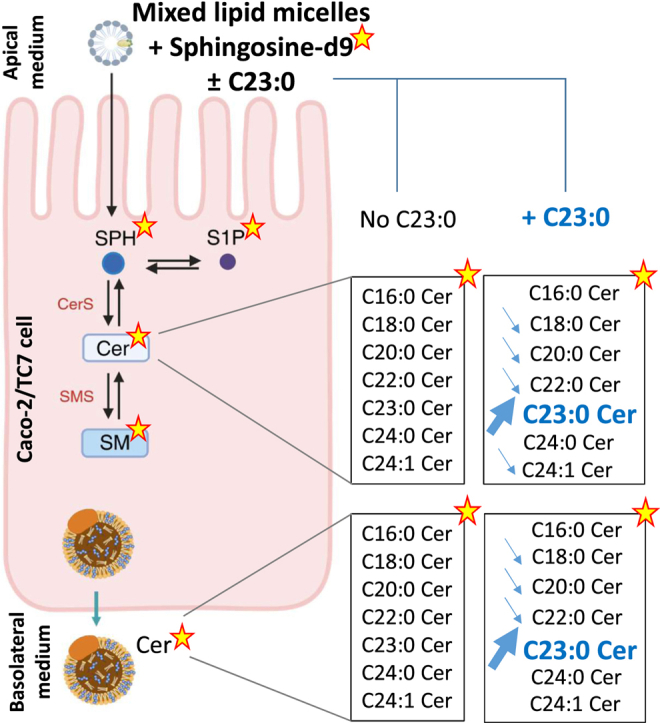

Chronic overconsumption of dietary lipids, mostly constituted of triglycerides (TG), contributes to the dysregulation of lipid metabolism in the small intestine (1), which in turn impacts the secretion of lipids in intestinally derived chylomicrons (2) and leads to postprandial hyperlipidemia. Sphingolipids (SL), particularly ceramides (Cer), play contributing roles in the development of metabolic disorders (3) and cardiovascular diseases (4) and can be modulated by pharmacological treatments and nutritional interventions. Among SL, sphingomyelin (SM) is the main circulating SL class (5) and an important component of the surface monolayer of lipoproteins and can also be found in the diet as one of the main polar lipids constitutive of the mammalian milk fat globule membrane (6, 7). Structurally, SM is composed of a phosphorylcholine head group linked to a Cer, composed of a sphingosine (SPH) linked to a fatty acid (FA) by an amide bond. SPH constitutes the backbone of SL and the most abundant one in mammals is SPH d18:1 (8). There is evidence that milk polar lipids (MPLs), notably via SM, may modulate intestinal lipid absorption and inflammation (9, 10). In addition, milk SM proved effective for lowering plasma cholesterol levels in high-fat fed mice (11). In overweight women at risk of cardiovascular diseases, 1-month daily supplementation with MPLs lowered postprandial concentrations of TG, cholesterol, and SM in intestine-derived chylomicrons (12, 13). Therefore, targeting the absorption of dietary SM in enterocytes may be an indirect way to control circulating lipid levels and thereby contribute to prevent or handle nutrition-driven chronic diseases, such as obesity and associated metabolic disorders. The vast majority of knowledge about the intestinal absorption of SM comes from an early pioneering study of Nilsson conducted in rats with radiolabeled SL (14), which has been extensively summarized in reviews (10, 15). Results showed that in lymph-duct cannulated rats, SM from ox brain was not directly absorbed but was sequentially hydrolyzed in the intestine by alkaline sphingomyelinase to phosphorylcholine and Cer and further hydrolyzed by a neutral ceramidase to SPH and unesterified FA, both ultimately absorbed and metabolized. Unanswered questions remain regarding the mechanisms underlying the metabolism of SPH and FA taken up by enterocytes and the secretion of SL and TG. Does the exogenous SPH-induced modified pool of SL in enterocytes result from the uptake of SPH from the apical membranes and/or from endogenous SL biosynthesis? Are milk SM-derived very-long-chain FAs incorporated into complex SL and secreted in the basolateral medium of enterocytes?

The objective of the present study was to investigate the uptake and endogenous metabolism of hydrolysis products of milk SM, i.e., SPH and milk SM-specific FA in an in vitro model of enterocytes, as well as the secretion of TG and main SL. To mimic micelles formed postprandially after a milk SM-enriched meal, mixed lipid micelles were prepared (16) and enriched with SPH, the main sphingoid base of milk SM (8), with or without tricosanoic acid (C23:0), a very-long odd-chain saturated FA originated from dairy fats (17, 18) and one of the main FA specific of milk SM (6, 19) but barely present in TG. In addition, because C23:0 is an odd-chain FA present only at trace levels in human cells, it can be easily tracked within cells. Caco-2/TC7 differentiated cells, cultured on semipermeable inserts, were used as an in vitro enterocyte model for evaluating intestinal lipid absorption (20, 21), independently of gut-brain and gut-liver communications and immune regulation. The use of a stable isotope-labeled SPH combined with analyses of SM, Cer, and main sphingoid bases by tandem mass spectrometry (MS/MS) allowed to provide quantitative data of SPH-based SL in enterocytes. Altogether, analyses of SM and Cer molecular species modified in enterocytes by SPH-enriched mixed micelle (MM) highlighted the importance of studying FA specific of dietary milk SM in order to decipher the specific impact of these FAs on SL metabolism and secretion.

## Materials and methods

### Reagents

Cell culture reagents were obtained from Life Technologies SAS (Villebon sur Yvette, France). Bovine milk SM and SPH (d18:1), N-heptadecanoyl-D-erythro-sphingosine (d18:1/17:0 Cer) and N-palmitoyl (d31)-D-erythro-sphingosylphosphorylcholine (d18:1/16:0-d31 SM), D-erythro-sphingosine-d7, and d17:1 sphingosine-1-phosphate (S1P) were from Avanti Polar Lipids (Alabaster, AL). 2-oleoyl glycerol was from Cayman Chemical (Ann Arbor, MI), and D-erythro-sphingosine d9 (SPH-d9) was from Matreya LLC (State College, PA). All other chemicals were from Sigma-Aldrich (Saint-Quentin-Fallavier, France).

### Cell culture

Caco-2/TC7 intestinal cells, kindly provided by Dr Rousset (Paris, France), were derived from a human colon adenocarcinoma. Cells were routinely cultured on 75 cm^2^ flasks in high-glucose DMEM GlutaMAX medium supplemented with 20% fetal calf serum, 1% nonessential amino acids, and 1% penicillin/streptomycin and maintained under a 10% CO_2_ atmosphere at 37°C (20). After reaching 80% confluence, cells were detached with trypsin and seeded on permeable ThinCert-cell culture inserts at a density of 5 × 10^4^ cells/cm^2^ (3 μm pore size Polyester Membrane, Greiner Bio-One France, Courtaboeuf, France) in 6-well plates to reproduce the intestinal barrier. Cells were grown to confluence in complete medium for 1 week. Cells were then cultured in asymmetric conditions, with 1.5 ml serum-free medium in the upper compartment and 2 ml medium containing fetal calf serum in the lower compartment, until total differentiation (around 21 days after seeding). The medium was changed every 2–3 days. Cell passage numbers ranging between 39 and 44 were used for the experiments. Cell monolayer integrity was assessed by measuring Lucifer Yellow (100 μM) permeability from the apical compartment to the basolateral compartment 21 days after seeding (22). In preliminary experiments, less than 1% Lucifer Yellow was detected in the basolateral medium of Caco-2/TC7 cells. Prior to the start of treatment, cells were incubated overnight with serum-free complete medium in both compartments.

### Preparation of mixed lipid micelles and incubation with cells

Mixed micelles (MM) were prepared in the presence of sodium taurocholate according to Chateau *et al.* (16). The final MM consisted of oleic acid (0.5 mM), 2-oleoyl glycerol (0.2 mM), soybean phosphatidylcholine (0.4 mM), L-α-lysophosphatidylcholine (0.2 mM), cholesterol (0.05 mM), and sodium taurocholate (2 mM) in serum-free medium supplemented with 1% nonessential amino acids, 1% pyruvate, and 1% antibiotics. Some MMs were enriched with SPH (0.05 mM) (MM+SPH) or milk SM-typical FA (C23:0) (0.05 mM) (MM+C23:0) or SPH and C23:0 (MM+SPH+C23:0), added at the same time as other lipids. Similar experiments were performed with stable isotope–labeled SPH-d9. In addition, other FAs (C18:0 or C22:0) instead of C23:0 were added to MM enriched with SPH-d9 to evaluate the specificity of the effects. Appropriate lipid stock solutions were added in sterile glass tubes and dried under a stream of nitrogen. Lipids were then dissolved in serum-free medium containing 24 mM taurocholate, vortexed for 10 min, and dispersed in an ultrasonic bath at 37°C for 10 min to obtain lipid micelles before adjusting the volume to the desired concentrations. Following apical incubation of MM, basolateral media and cells were separately collected and stored at −80°C for further analysis. The cell monolayer was recovered by scraping after two times washing with serum-free medium.

### Particle size measurement

MM and chylomicron-like particles secreted in Caco-2/TC7 basolateral media were characterized by dynamic light scattering using a Zetasizer NanoS (Malvern, UK). All measurements were performed in triplicate in DMEM medium at 37°C with an equilibration time of 180 s using 0.84 cP and 1.445 as viscosity and refractive index, respectively. Prepared MM enriched or not with SPH contained structures of micellar size with mean hydrodynamic diameters of 4.1 ± 0.2 nm and 4 ± 0.3 nm, respectively (n = 3).

### Quantification of triglycerides

TG concentrations into the basolateral media were measured by a fluorimetric TG assay kit according to the manufacturer's instruction (Abcam, Cambridge, United Kingdom). Preliminary time course experiments showed that apical addition of MM to Caco-2/TC7 cells led to secretion of TG in the basolateral media, increasing gradually from 4 to 16 h with no further significant increase at 20 h (supplemental Fig. S1). Therefore, cells were incubated for 16 h with the different MM preparations into the apical compartment.

### RNA extraction and quantitative real-time PCR

Total RNA was extracted from Caco-2/TC7 cells with TRI Reagent (Sigma-Aldrich, Saint-Quentin-Fallavier, France) following manufacturer’s instructions and resuspended in RNase-free water. RNAs were quantified with NanoDrop One (Thermo Fisher Scientific, Waltham, MA), and RNA samples with A260/280 ratio between 1.7 and 2.1 were considered of good purity. Reverse transcription was performed using PrimeScript RT reagent kit (Ozyme, Saint Quentin en Yvelines, France) with 1 μg of RNA. Real-time PCR assays were performed using a Rotor-Gene Q (Qiagen, Hilden, Germany) and SYBR qPCR Premix Ex Taq (Tli RNaseH Plus) reagents. Relative mRNA expressions of intestinal Cer synthases (CerSs) and SM synthases (SMSs) were analyzed and normalized to the housekeeping gene TBP (TATA box binding protein) in each sample. PCR primers are listed in supplemental Table S1.

### Analyses of **sphingosine and sphingosine-1-phosphate** by LC-MS/MS

Cells (200 μg total proteins) and basolateral media (200 μl) were precipitated with 500 μl methanol containing D-erythro-sphingosine-d7 and d17:1 S1P as internal standards and 500 μl 2% H_3_PO_4_ (v/v) (23). Following centrifugation, the supernatant was transferred to mixed-mode cation-exchange solid phase extraction SPE columns (Oasis MCX, Waters Corp, Milford, MA). Columns were washed with 2% formic acid and eluted successively with 0.2% formic acid in methanol and 2% NH_4_OH in methanol. Lipid extracts were evaporated with nitrogen and redissolved with 500 μl of 0.2% formic acid in 37.5% acetonitrile. LC-MS/MS analysis was performed using two LC pumps (LC20AD) and an autosampler (SIL20AC) (Prominence Liquid Chromatograph, Shimadzu©, Kyoto, Japan) coupled to an API 4500 QTRAP (Sciex Applied Biosystems, Toronto, Canada) operated in the multi reaction monitoring (MRM) mode. Positive ionization mode was used for SPH and negative ionization mode for S1P. Reverse phase LC was performed on a C_8_ column (Uptisphere® 120 Å, 3 μm, 2.1 mm × 50 mm, Interchim©, Montluçon, France) maintained at 25°C (phase A: 0.2% formic acid in H_2_O, phase B: 0.2% formic acid in acetonitrile). The following gradient was run at a flow rate of 0.4 ml/min. The gradient was initiated at 37.5% of phase A, 0–4 min: 37.5%–50% B, 4–8 min: 50% B, 8–8.5 min: 50%–75% B, 8.5–9.5 min: 75% B, 9.5–10 min: 75%–37.5% B, and 10–13 min: 37.5% B. Twenty-five microliter was injected, and the total run time was 13 min. Quantification of SPH (d18:1) and S1P (d18:1) was achieved by a five-point external standard calibration and linear regression using the Analyst software version 1.6.2. The interassay coefficients of variation were 8.9% and 21.1% for d18:1 SPH and d18:1 S1P, respectively.

### Analyses of sphingomyelins and ceramides by MS/MS

A one-phase lipid extraction with chloroform/methanol (1:2) was performed on Caco2/TC7 cells (150 μg total proteins), and basolateral media (150 μl). (d18:1/17:0) Cer and (d18:1/16:0-d31) SM were added as internal standards prior to lipid extraction. The lipid-containing phase was evaporated with nitrogen, and dried lipid extracts were stored at −20°C. Dried lipid extracts were dissolved in chloroform/methanol (1:2) and saponified with 1 M potassium hydroxide in methanol to remove glycerolipids. Samples were then incubated for 2 hours at 37°C and neutralized with acetic acid. Samples were purified on a SPE C18 column (Bond Elut) by a double extraction using methanol/H_2_O (1:1), and elution of Cer and SM was done with chloroform/methanol (1:2) (13). Samples were evaporated with nitrogen, resolubilized in chloroform/methanol (2:1), and analyzed by direct flow injection on a triple-quadrupole mass spectrometer (API 4500 QTRAP MS/MS; Sciex Applied Biosystems, Toronto, Canada) in the positive ionization mode using the MRM mode. Cer and SM as well as Cer-d9 and SM-d9 were quantified separately with a flow rate of 200 μl/min (analysis time of 3 min). The annotations of Cer and SM molecular species were based on the following assumptions: *i*) SPH d18:1 was the most abundant sphingoid base (24) (it is likely that other isomers with the same m/z were present (25) but not shown for the sake of clarity) and *ii*) C23:0 was preferentially incorporated. It should be specified that the annotation was not based on the shorthand notation for SL derived from mass spectrometry analyses (26) but from assumptions considering the sphingoid base SPH (d18:1) and FA (either C23:0, C22:0 or C18:0) that were added in MM to the apical medium of Caco-2/TC7 cells. Details of the MRM transitions of each of 16 quantified Cer and SM molecular species are shown in supplemental Table S2. The concentration of each Cer and SM molecular species containing SPH (d18:1) was calculated from the ratio of its signal to that of the corresponding internal standard. Total Cer and SM corresponded to the sum of the various species. The interassay coefficients of variation were 15.7% and 6% for total Cer and total SM, respectively.

### Analyses of C23:0 Cer and C23:0 SM in human intestine–derived chylomicrons

Complementary analyses of SL species were performed on chylomicrons previously collected in the randomized controlled trial “VALOBAB-C”. This clinical trial registered at Clinical Trials (NCT02099032) was approved by the Scientific Ethics Committee of Lyon Sud Est-IV and ANSM (French Agency for the Safety of Health Products) and conducted in accordance with the principles of the Helsinki declaration. Informed written consent was obtained from all subjects. Details of the VALOBAB-C trial have been published previously (12). Briefly, chylomicron-rich fractions were collected from plasma of postmenopausal women before and after a standardized lunch including cream cheese almost devoid of milk SM or enriched with 3–5 g of MPL (i.e., 0.8–1.2 g of milk SM) via a butter serum concentrate. Major SM and Cer species carrying even-chain FA were previously analyzed (13), and a specific analysis of C23:0 SM and C23:0 Cer species, normalized by the TG content of chylomicrons, was done in the framework of the present study.

### Statistical analysis

All data, presented as means ± standard error of the mean, were analyzed with Graph Pad Prism Software (version 7, San Diego, CA). Comparison between two groups was performed using unpaired Student's *t* test, and comparisons of multiple groups were analyzed by ordinary one-way ANOVA followed by Tukey's post hoc test.

## Results

### Incubation of Caco2/TC7 cells with mixed micelles enriched with sphingosine and/or C23:0 led to a similar secretion of triglycerides than cells incubated with mixed micelles

**Fig. 1:**
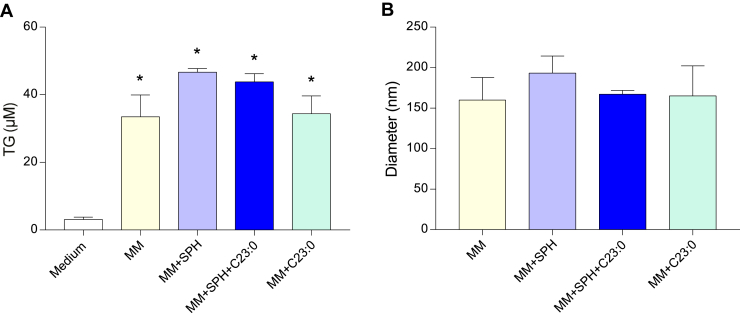
Triglyceride concentrations and hydrodynamic diameters of chylomicron-like particles in the basolateral medium of Caco-2/TC7 cells. TG concentrations (A) and hydrodynamic diameters of chylomicron-like particles (B) in Caco-2/TC7 cells incubated for 16 h with medium alone (Medium), mixed micelles (MM), mixed micelles enriched with either sphingosine (MM + SPH), sphingosine + C23:0 (MM + SPH + C23:0), or C23:0 (MM + C23:0). Data represent the mean ± SEM of three independent experiments realized in triplicate. Asterisks represent a significant difference compared to medium (∗*P* < 0.05).

Compared to cells incubated for 16 h with medium alone devoid of lipids, mean TG concentrations were increased by 11-fold in the basolateral medium of cells enriched with MM composed of oleic acid, 2-oleoyl glycerol, phosphatidylcholine, lysophosphatidylcholine, cholesterol, and sodium taurocholate (Fig. 1A). The enrichment of MM with either SPH, (SPH + C23:0), or C23:0 increased TG secretion to the same extent, indicating that the supply of 0.05 mM SPH and/or 0.05 mM C23:0 did not alter TG secretion. In addition, particles present in the basolateral medium of cells incubated with different MM had a hydrodynamic diameter ranged between 160 and 193 nm, reflecting the secretion by cells of TG-rich lipoproteins. The size of these TG-rich lipoproteins was not modified by the lipid composition of MM added to cells (Fig. 1B).

### Enrichment of mixed micelles with sphingosine and C23:0 increased the uptake of C23:0 and its incorporation into Cer and SM

**Fig. 2:**
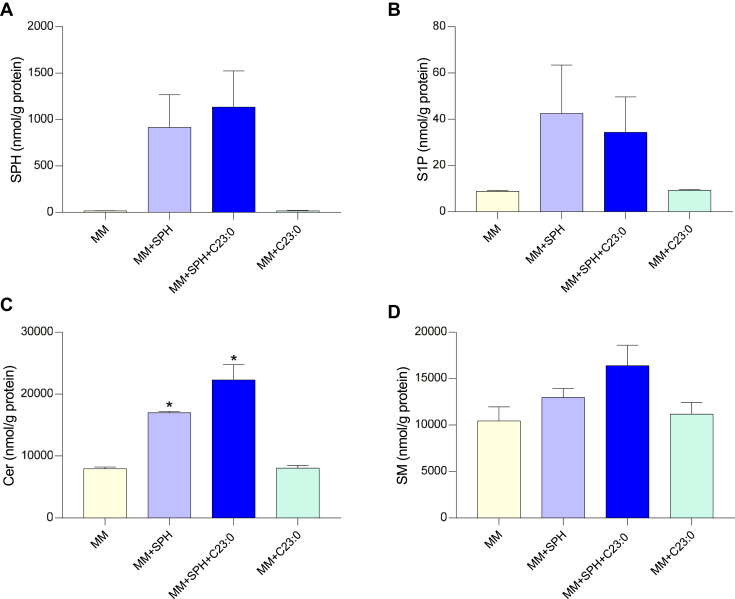
Total concentrations of sphingosine, sphingosine-1-phosphate, ceramides, and sphingomyelins in Caco-2/TC7 cells. Sphingosine (SPH) (A), sphingosine-1-phosphate (S1P) (B), ceramides (Cer) (C), and sphingomyelins (SM) (D) concentrations in cells incubated for 16 h with mixed micelles (MM), mixed micelles enriched with either sphingosine (MM + SPH), sphingosine + C23:0 (MM + SPH + C23:0), or C23:0 (MM + C23:0). Data expressed as nmol/g protein, represent the mean ± SEM of three independent experiments. Asterisks represent a significant difference compared to MM-enriched cells (∗*P* < 0.05).

**Fig. 3:**
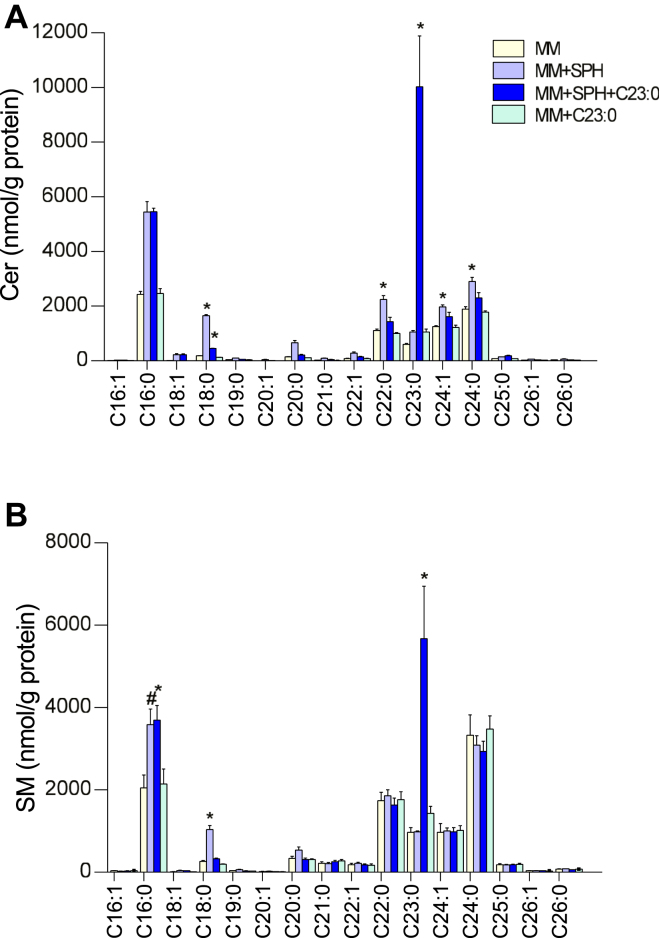
Molecular species of ceramides and sphingomyelins in Caco-2/TC7 cells. Ceramides (Cer) (A) and sphingomyelins (SM) (B) molecular species concentrations in cells incubated for 16 h with mixed micelles (MM), mixed micelles enriched with either sphingosine (MM + SPH), sphingosine + C23:0 (MM + SPH + C23:0), or C23:0 (MM + C23:0). Cer and SM molecular species annotations were based on the assumption that sphingosine d18:1 is the major sphingoid base. Data expressed as nmol/g protein, represent the mean ± SEM of three independent experiments. Asterisks represent a significant difference compared to MM-enriched cells (∗*P* < 0.05, #*P* = 0.06).

To determine the impact of SPH on SL within Caco-2/TC7 cells, SPH and S1P as well as molecular species constitutive of total Cer and SM were analyzed (Fig. 2). Compared to cells incubated with MM, higher concentrations of SPH and S1P were observed in cells incubated with SPH-enriched micelles, regardless of the presence or not of C23:0, but differences did not reach statistical significance due to interexperimental variability (Fig. 2A, B). Apical supply of cells with SPH-enriched or (SPH+C23:0)-enriched micelles did not significantly modify total SM concentrations but led to higher concentrations of total Cer within cells (Fig. 2C, D). Adding C23:0 alone in MM had no effect on SPH, S1P, SM, and Cer concentrations in cells compared to cells incubated with MM. Quantification of SL molecular species containing even-chain FA species but also odd-chain FA showed differences between Cer and SM profiles in (MM + SPH)-loaded cells compared to MM-loaded cells (Fig. 3). In cells incubated with SPH-enriched MM, most major molecular species of Cer increased, i.e., C16:0 (2.2-fold), C18:0 (9-fold), C22:0 (2-fold), C24:1 (1.6-fold), and C24:0 (1.5-fold) Cer. The coaddition of C23:0 and SPH in MM led to a 16.5-fold increase of C23:0 Cer and increased C16:0 Cer (2-fold) and C18:0 Cer (2.5-fold) in cells, while the incorporation of C23:0 alone in MM had no effect on any Cer species (Fig. 3A). Although total SM concentrations did not differ between cells enriched with different micelles, C18:0-SM concentration was higher (3.9-fold) in (MM+SPH)-enriched cells compared to MM-enriched cells, and C16:0-SM also tended to be higher (1.8-fold, *P* = 0.061). Incubation of cells with (SPH+C23:0)-enriched micelles led to a 5.9-fold increase of C23:0 SM and doubled C16:0 SM in cells, while C23:0-enriched micelles had no effect on SM molecular species (Fig. 3B). In summary, Caco-2/TC7 cells incubated for 16 h with SPH-enriched MM had higher total Cer concentrations than MM-loaded cells, and coaddition of C23:0 in SPH-enriched MM preferentially increased C23:0 Cer and C23:0 SM species.

### Effects of mixed micelles enriched with sphingosine and C23:0 on intestinal enzymes involved in Cer and SM synthesis

**Fig. 4:**
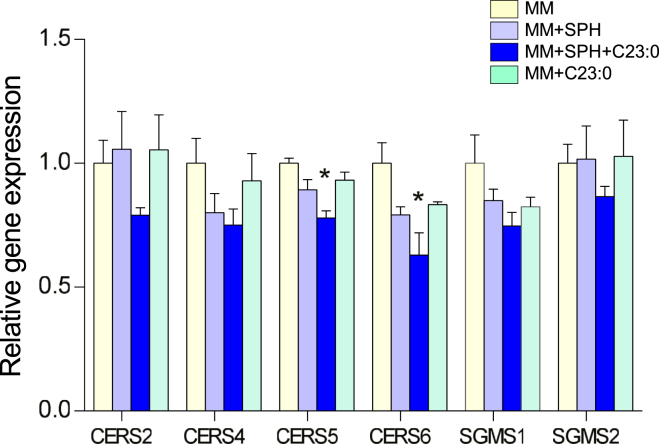
Ceramide and sphingomyelin synthase gene expression. Gene expression of ceramide synthase 2 (CERS2), ceramide synthase 4 (CERS4), ceramide synthase 5 (CERS5), ceramide synthase 6 (CERS6), SM synthase 1 (SGMS1), and SM synthase 2 (SGMS2) in Caco-2/TC7 cells incubated for 16 h with mixed micelles (MM), mixed micelles enriched with either sphingosine (MM + SPH), sphingosine + C23:0 (MM + SPH + C23:0), or C23:0 (MM + C23:0). Values for each gene were normalized to TBP mRNA. Data are expressed as fold-change versus MM-incubated cells and represent the mean ± SEM of three independent experiments. Asterisk represents a significant difference compared to MM incubation (∗*P* < 0.05).

Whether 16 h of incubation with MM ± SPH and/or C23:0 modified the expression of genes involved in Cer and SM synthesis was explored in Caco-2/TC7 cells. Main CerSs expressed in human intestine (27) were analyzed. Gene expression levels of CerS5 and CerS6, which synthesize mainly C14-C16 Cer, decreased in cells incubated with (SPH+C23:0)-enriched MM compared to MM, whereas there was no significant changes of the gene expression levels of CerS2, which synthesizes Cer containing C20-C26 FAs, and of CerS4, which synthesizes Cer containing C18-C20 FAs. No differences were detected in the expression of the genes coding for the two SMS (SGMS1 and SGMS2) catalyzing the synthesis of SM from Cer, after incubation of cells with the different MM (Fig. 4).

### Stable isotope-labeled sphingosine incorporated into mixed micelles is absorbed and partly converted to S1P, Cer, and SM in Caco-2/TC7 cells

**Fig. 5:**
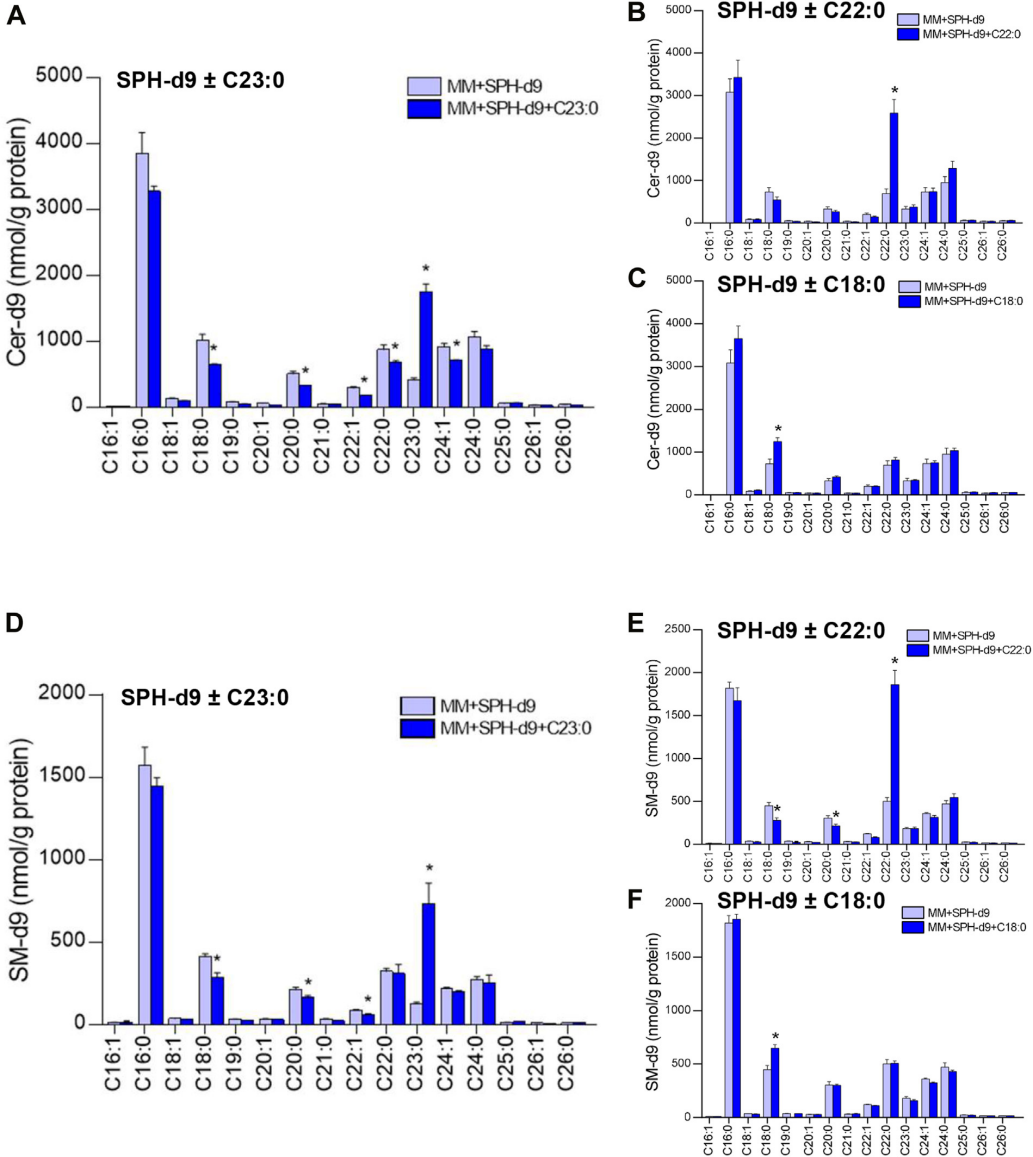
Molecular species concentrations of deuterated ceramides and deuterated sphingomyelins in Caco-2/TC7 cells. Deuterated ceramides (Cer-d9) (A, B, and C) and deuterated sphingomyelins (SM-d9) (D, E, and F) molecular species concentrations in cells incubated for 16 h with mixed micelles enriched with sphingosine-d9 (MM + SPH-d9) or with sphingosine-d9 + C23:0 (MM + SPH-d9 + C23:0) (A, D) or with sphingosine-d9 + C22:0 (MM+SPH-d9 + C22:0) (B, E) or sphingosine-d9 + C18:0 (MM + SPH-d9 + C18:0) (C, F). Cer-d9 and SM-d9 molecular species annotations were based on the assumption that sphingosine d18:1 is the major sphingoid base. Data expressed as nmol/g protein, represent the mean ± SEM of one experiment realized in quadruplicate. Asterisks represent a significant difference between MM+SPH and MM+SPH+FA (∗*P* < 0.05).

In order to track the uptake and metabolism of SPH added in the apical medium of cells and to distinguish between SL synthesized from exogenous SPH and endogenous SL, similar experiments were performed with a stable isotope-labeled SPH-d9. Apical supply of MM containing either SPH-d9 or (SPH-d9+C23:0) to cells led to an appearance of SPH-d9 and S1P-d9 within cells (Table 1), indicating an absorption of SPH through the intestinal villi membranes and a conversion of SPH to S1P by SPH kinase in both supply conditions. It also resulted in an enrichment of cells with Cer-d9 and SM-d9 (Table 1), showing that absorbed SPH-d9 was converted to Cer-d9 by CerS and further to SM-d9 by SMS. Regarding the distribution of the FA within Cer-d9 and SM-d9, all molecular species were detected in significant amounts in cells loaded with both types of SPH-d9-enriched MM (Fig. 5). However, (MM+SPH-d9+C23:0)-incubated cells showed higher C23:0 Cer-d9 (4.3-fold) and lower C18:0 (1.6-fold), C20:0 (1.6-fold), C22:1 (1.6-fold), C22:0 (1.3-fold), and C24:1 (1.3-fold) Cer-d9 concentrations compared to (MM+SPH-d9)-loaded cells (Fig. 5A). Among SM-d9 molecular species, C23:0 SM-d9 was higher (5.8-fold), while C18:0, C20:0, and C22:1 SM-d9 were lower (1.4, 1.3, and 1.5-fold, respectively) in cells incubated with (SPH-d9+C23:0)-enriched MM compared to those incubated with SPH-d9-enriched MM (Fig. 5D). Parallel quantification of unlabeled Cer and SM in cells did not show any difference of SL molecular species concentrations between cells incubated with either SPH-d9-enriched MM or (SPH-d9+C23:0)-enriched MM. Compared to MM-incubated cells, unlabeled C16:0 Cer and C16:0 SM decreased in (MM+SPH-d9) and (MM+SPH-d9+C230)-incubated cells (supplemental Fig. S2). To determine whether the preferential incorporation of C23:0 into Cer-d9 and SM-d9 in (MM+SPH-d9+C23:0)-incubated cells was specific to this FA or not, cells were also incubated with (MM+SPH-d9) and either C22:0, other very-long-chain FA present in significant amounts in bovine milk SM (24%) or with C18:0, a FA present at trace levels in bovine milk SM (1.5%). Compared to (MM+SPH-d9)-loaded cells, (MM+SPH-d9+C22:0)-incubated cells showed higher concentrations of C22:0 Cer-d9 and C22:0 SM-d9 (3.7-fold for both) (Fig. 5B, E), while C18:0 Cer-d9 and C18:0 SM-d9 concentrations increased to a lower extent in (MM+SPH-d9+C18:0)-incubated cells (1.7-fold and 1.4-fold, respectively) (Fig. 5C, F). In summary, the incubation of Caco-2/TC7 cells with MM containing SPH-d9 and milk SM-specific very-long-chain FA, i.e., C23:0 or C22:0, resulted in an enrichment of Cer-d9 and SM-d9 with the respective FA, at the expense of other FA.

**Table 1:** Sphingolipids and sphingoid bases in Caco-2/TC7 cells and basolateral medium of cells incubated with deuterated sphingosine-enriched lipid micelles

Sphingolipids	MM + SPH-d9	MM + SPH-d9 + C23:0
	pmol/well	
Cells		
SPH-d9	53.2 ± 2.7	58.2 ± 2.6
S1P-d9	7.3 ± 0.3	5.4 ± 0.9
d18:1 Cer-d9	2,850 ± 218	2,880 ± 125
d18:1 SM-d9	1,039 ± 43	1,185 ± 108
Basolateral medium		
SPH-d9	ND	ND
S1P-d9	ND	ND
d18:1 Cer-d9	1,159 ± 62	1,228 ± 15
d18:1 SM-d9	ND	ND

Deuterated SPH (SPH-d9), deuterated S1P (S1P-d9), deuterated Cer (d18:1 Cer-d9), and deuterated SM (d18:1 SM-d9) in cells and basolateral medium of Caco-2/TC7 cells incubated for 16 h with MM enriched with SPH-d9 (MM + SPH-d9) or with SPH-d9 and C23:0 (MM + SPH-d9 + C23:0). MM preparation containing 75 nmol SPH-d9 was added in 1.5 ml apical medium. Data expressed as pmol/well, represent the mean ± SEM of one experiment realized in quadruplicate. No significant difference was observed between sphingolipids in (MM + SPH-d9) and (MM + SPH-d9 + C23:0) groups.

MM, mixed micelles; ND, not detectable; SPH, sphingosine; SPH-d9, deuterated sphingosine.

### Stable isotope-labeled sphingosine is partly secreted as Cer in the basolateral medium of Caco-2/TC7 cells

**Fig. 6:**
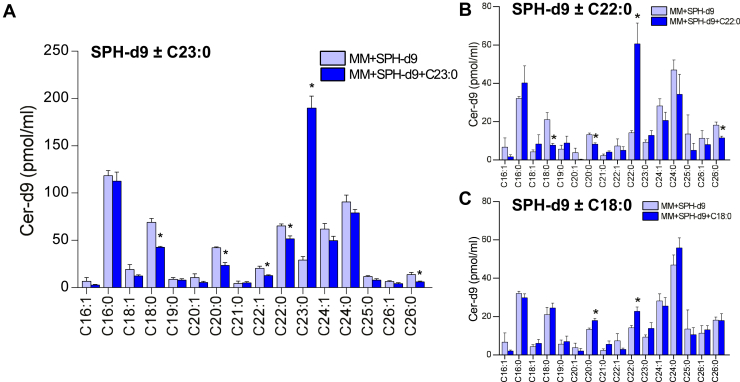
Molecular species concentrations of deuterated ceramides in the basolateral medium of Caco-2/TC7 cells. Deuterated ceramides (Cer-d9) molecular species concentrations in the basolateral medium of Caco-2/TC7 cells incubated for 16 h with mixed micelles enriched with sphingosine-d9 (MM + SPH-d9) or with sphingosine-d9 + C23:0 (MM + SPH-d9 + C23:0) (A) or with sphingosine-d9 + C22:0 (MM + SPH-d9 + C22:0) (B) or sphingosine-d9 + C18:0 (MM + SPH-d9+C18:0) (C). Cer-d9 molecular species annotations were based on the assumption that sphingosine d18:1 is the major sphingoid base. Data expressed as pmol/ml, represent the mean ± SEM of one experiment realized in quadruplicate. Asterisks represent a significant difference between MM + SPH and MM + SPH+FA (∗*P* < 0.05).

After 16 h incubation of cells with MM containing either SPH-d9 or (SPH-d9 + C23:0), deuterated Cer (Cer-d9) appeared in the basolateral medium showing that SPH-d9 was not only absorbed and partly incorporated into Cer-d9 in cells but also partly secreted as Cer (Table 1). SPH-d9, S1P-d9, and SM-d9 were not present at detectable levels in the basolateral medium of cells incubated in the presence of MM enriched with SPH-d9 or with SPH-d9 and C23:0 (Table 1). Basolateral secretion of Cer-d9 represented around 2% of SPH-d9 initially incubated with MM in the apical medium, regardless of the presence or not of C23:0, and SPH-d9 was incorporated in all Cer-d9 molecular species (Fig. 6A). Note that 6.5-fold higher concentrations of C23:0 Cer-d9 were found in the basolateral medium of (MM + SPH-d9 + C23:0)-enriched cells compared to those in the medium of (MM + SPH-d9)-enriched cells while C18:0, C20:0, C22:1, and C22:0 Cer-d9 concentrations were lower (1.6, 1.8, 1.6, and 1.3-fold, respectively) (Fig. 6A). Regarding the addition of other FA to MM enriched with SPH-d9, (MM+SPH-d9+C22:0)-incubated cells showed higher C22:0 Cer-d9 in the basolateral medium (4.3-fold) (Fig. 6B), while C18:0 Cer-d9 did not increase in the basolateral medium of (MM+SPH-d9+C18:0)-incubated cells (Fig. 6C) compared to (MM+SPH-d9)-loaded cells. Altogether, the addition of C23:0 or C22:0 to SPH-d9-enriched MM resulted in the basolateral secretion of C23:0 Cer-d9 or C22:0 Cer-d9, respectively.

### Milk SM increased C23:0 Cer/TG ratio in human chylomicrons

In complementary analyses of chylomicrons collected in the VALOBAB trial, we calculated the postprandial variation in composition, i.e., the difference between chylomicrons collected at 4 h [transit time of milk SM/Cer in the small intestine (12, 28)] after lunch minus those before lunch. This allowed to test the response difference according to the lunch being enriched or not with MPL (thereby providing 0.8–1.2 g milk SM via a butterserum concentrate or not). The postprandial increase in C23:0 Cer/TG ratio was higher in chylomicrons from the groups consuming 3 g MPL to 5 g MPL (+88 ± 18 nmol C23:0 Cer/mmol TG, n = 11) compared to the control group consuming a lunch not enriched in MPL (+10 ± 37 nmol C23:0 Cer/mmol TG, n = 6) (*P* = 0.049). Regarding SM, no change in the postprandial C23:0 SM/TG ratio was observed between these groups.

## Discussion

There is general consensus that SM is sequentially hydrolyzed to Cer and further to SPH and unesterified FA in the small intestine, based on studies performed in rats using ox brain SM (14). The present results give a more detailed picture of the complex metabolism of dietary SM-derived SPH in an in vitro model of enterocytes, independently of bacteria SL metabolism and immune modulation (29). Although odd-chain FAs have been poorly studied for many years due to their very low abundance in plasma and dairy fats, the present work also highlights the uptake of C23:0, a specific very-long-chain FA of milk SM, and its incorporation into Cer and SM, when combined to SPH.

In the present study, Caco-2 cell line was chosen to determine the uptake and metabolism of SPH in enterocytes because it is the most widely used in vitro model for studies of intestinal absorption and metabolism (30, 31). Although originally derived from a human colonic carcinoma, Caco-2 intestinal cells are indeed able to spontaneously differentiate in long-term culture and to polarize, when seeded on semipermeable membranes, in a monolayer of cells expressing several morphological and functional characteristics of the mature enterocyte. The TC7 clone isolated from a late passage of the parental Caco-2 cell line has been shown to consist of a more homogeneous population and proved to be particularly relevant for lipid absorption studies (20, 21). Incubation of Caco-2/TC7 cells with MM or digested milk does not affect cellular permeability and monolayer cell integrity assessed by occludin immunostaining as previously shown by our research group (22, 32). Regarding the hydrodynamic diameter of MM, their size (4 nm) was in the range of micelle size reported by other authors (33, 34) and was not modified by the presence of SPH. The present in vitro model of enterocytes enabled to monitor the accumulation of TG in the basolateral medium, independently of TG hydrolysis by lipoprotein lipase that occurs in vivo. Following supply of MM to Caco-2/TC7 cells, basolateral secretion of TG as well as formation of TG-rich lipoproteins with diameters of 150–200 nm were observed in agreement with previous studies (16, 35). No decreased TG secretion was evidenced in cells incubated with SPH-enriched MM compared to MM-incubated cells, in line with results reporting similar amounts of TG in the lymph of rats administrated a triolein emulsion or a (triolein + milk SM) emulsion (36). Our present in vitro results suggest that our previous in vivo results showing lowered postprandial concentrations of TG in chylomicrons from postmenopausal women supplemented with MPL (12) may be in favor of an enhanced chylomicron clearance rather than a reduced chylomicron TG secretion.

The results of the present study add new information on the uptake by enterocytes of SPH and on its metabolism. Our quantitative data on the concentrations of SL present in Caco2/TC7 cells following the addition of SPH-enriched MM indicate that exogenous amphiphilic SPH may have been absorbed through the intestinal membrane, partly absorbed as free SPH, partly converted to S1P and incorporated into Cer, without any significant change of total SM in Caco-2/TC7 cells. It is likely that the interassay variability and very low concentrations of free sphingoid bases (37) may explain the absence of statistical significance. Exogenous SPH was also recovered as Cer in the basolateral medium of cells, although to a minor extent (2%). Present results are consistent with initial studies from Ake Nilsson on the absorption of SM in the gut of rats (14). The latter showed that feeding lymph duct cannulated rats with a triolein emulsion together with tritiated SPH led to an absorption of SPH and to its reincorporation into Cer and SM in the mucosal cells. Recovery of radioactivity into lymph lipids was very low (7%), two-third into glycerides + FA, one-third into Cer, and 5% into SM. Tritiated Cer represented 2.6% of dietary SPH given to rats, in accordance with 2% Cer found in the basolateral medium of Caco-2/TC7 cells following SPH supply. Additional results from Schmelz *et al.* (38) showed that tritium from radiolabeled SPH was rapidly recovered in intestinal segments of mice and distributed into Cer, SM, and glycolipids (glucosylCer and lactosylCer), in line with the present results on increased total Cer and SM concentrations found in cells incubated with SPH-enriched micelles. GlucosylCer was not analyzed in our experiments because Cer conversion to complex glycosphingolipids was beyond the scope of the current study. However, a detailed characterization of complex SL definitely deserves further investigation.

In addition, the use of a stable isotope-labeled SPH in the present study gave information on the source of SL present in cells supplied with SPH-containing micelles and enabled to differentiate SL of exogenous source from endogenous SL pool. Our results demonstrate that the accumulation of free SPH, S1P, and total Cer in (MM + SPH)-incubated cells mostly resulted from the uptake of exogenous SPH but not from the SL pool or de novo SL synthesis. Furthermore, the targeted analyses of SM and Cer molecular species highlighted the increases of most major molecular species of Cer, C16:0, C18:0, C22:0, C24:1, and C24:0 Cer as well as the increase of C18:0 SM in (MM+SPH)-incubated cells compared to MM-incubated cells. The unlabeled C16:0 Cer molecular species, only reflecting endogenous C16:0 Cer, decreased in (MM + SPH-d9 ± C23:0)-incubated cells compared to MM-incubated cells, suggesting an effect of exogenous SPH on Cer biosynthesis. Present data show a huge increase of C23:0 Cer and C23:0 SM in cells enriched with MM+(SPH+C23:0) compared to those incubated with MM+SPH, while the addition of C23:0 alone to MM had no effect on SM and Cer profiles. This supports that SPH, as a building block of SL and a limiting factor in SL biosynthesis, is required to get a significant incorporation of C23:0 into SL. C23:0 Cer was also the most abundant molecular species present in the basolateral medium of MM+(SPH+C23:0)-enriched cells. C23:0 was chosen as a FA supplied to MM in the present study because milk SM contains significant proportions of C23:0 (6, 19) together with high proportions of very-long-chain saturated FA (from C20:0 to C24:0) compared to egg or brain SM (8). These very-long-chain FA are mostly found in SL, while being present as traces in phospholipids and TG (39). Our results suggest that micelles enriched with both SPH and C23:0, such as found in vivo in postprandial micelles formed after milk SM ingestion, could impact the formation and FA distribution of endogenous Cer and might favor the secretion of chylomicrons enriched with C23:0 Cer. This assumption was reinforced with our complementary results showing that the addition of other very-long-chain present in milk SM, C22:0, also resulted in its preferential incorporation into cell and secreted Cer. The complementary genomics results show decreased gene expression levels of CerS5 and CerS6 in (MM+SPH+C23:0)-incubated cells, which were not associated with C16:0 Cer changes. Considering that gene expression levels of CerS did not always correlate with Cer molecular species levels in previous studies (27, 40, 41), we cannot exclude that either protein expression or the activity of the CerS was regulated posttranscriptionally.

Our results corroborate studies performed in lymph-cannulated rats showing that ingestion of an emulsion containing milk SM and phospholipids enhanced the lymph concentrations of Cer molecular species found in milk such as C16:0, C22:0, C23:0, and C24:0 Cer (36). Whether an increase of circulating C23:0 Cer has functional consequences remains to be elucidated.

Within intestinal cells, increases of Cer C23:0 in cells enriched with both SPH and C23:0 were accompanied by decreases of C18:0, C20:0, C22:1, C22:0, and C24:1 species. In the basolateral medium reflecting TG-enriched lipoproteins, higher C23:0 Cer concentration was accompanied by lower concentrations of C18:0, C20:0, C22:1, and C22:0 Cer. Considering that some of these Cer molecular species, i.e., C18:0, C20:0, and C24:1 Cer were reported to be deleterious species for metabolic and cardiovascular health (42), modulation of the FA profile of Cer by dietary means may represent an interesting approach. Previous studies indeed showed that high plasma concentrations of C16:0, C18:0, and C24:1 Cer were associated with poor cardiovascular outcomes and increased mortality (43), while positive associations between specific Cer species (C16:0, C22:0, C24:1 and C24:0) and the incidence of cardiovascular events were observed in patients from the PREDIMED trial (44). Accumulating evidence also suggests that intake of dairy products, which are typically high in SFA content, is inversely associated with type 2 diabetes (45, 46). Odd-chain SFAs (C15:0 and C17:0) and longer-chain SFAs (C20:0, C22:0, C23:0, and C24:0) were inversely associated with type 2 diabetes in the EPIC-InterAct case-cohort study, while even-chain SFAs (C14:0, C16:0, and C18:0) were positively associated with the incidence of type 2 diabetes (47).

Our group showed previously that 1-month consumption up to 5 g/day of MPL significantly decreased C22:0, C24:0, and C24:1 Cer species in intestine-derived chylomicrons of postmenopausal women at risk of CVD (13). In this trial, postprandial metabolic explorations were performed before and after nutritional intervention with MPL. In the context of the present work in Caco-2/TC7 cells with acute incubations of MM and the specific tracing of C23:0 SM and Cer, we performed a complementary analysis of postprandial chylomicrons from the VALOBAB trial, showing that C23:0 Cer/TG ratio increased more in chylomicrons from women who consumed a lunch enriched with MPL compared to chylomicrons from women who consumed a lunch without MPL. Such clinical results reinforce the relevance of results obtained in the present Caco-2/TC7 study. As the food matrix can modulate dietary lipid bioaccessibility and absorption (48, 49), it would now be relevant to study postprandial milk-specific SL from different dairy foods.

In conclusion, our original approach using stable isotope tracing provide a more detailed insight into the uptake and metabolism of dietary milk SM-derived SPH in enterocytes. In addition, our results demonstrate that C23:0 odd-chain long-chain FA, when combined with SPH, is preferentially incorporated into Cer and SM within enterocytes and into Cer that is found in the basolateral medium.

Altogether, dietary lipids providing precursor substrates for enterocyte SL metabolism, such as SPH and FA, are a critical factor controlling Cer concentrations both in the intestine tissues and blood circulation. Milk SL appear as promising dietary candidates in the restoration or the maintaining of metabolic health.

## Data availability

All data of the present study are contained in the manuscript and in the Supplementary figures. Data can be shared upon request by contacting Armelle Penhoat (armelle.penhoat@inserm.fr).

## Supplemental data

This article contains supplemental data.

## Conflict of interest

M.-C. M. received research funding from CNIEL, Sodiaal-Candia R&I, and Danone Nutricia Research that are not related to the present in vitro study. CNIEL was a partner of the VALOBAB project coordinated by M.-C. M. Other authors have no conflict of interest to declare.
