# Supplementary material for: Intestinal absorption of sphingosine: new insights on generated ceramide species using stable isotope tracing in vitro
Source: J Lipid Res. 2024 May 7;65(6):100557. doi: 10.1016/j.jlr.2024.100557 (PMC11179623; doi:10.1016/j.jlr.2024.100557)
Supplement: Supplemental data [file mmc1.docx]

SUPPLEMENTAL DATA:

**Intestinal absorption of sphingosine: new insight on generated ceramide species using stable isotope tracing *in vitro***

Catherine Calzada^1a^, David Cheillan^1,2a^, Nina Ritsch^1a^, Cécile Vors^1^, Annie Durand^1^, Sandra Pesenti^1^, Magali Pettazzoni^2^, Emmanuelle Meugnier^1^, Marie-Caroline Michalski^1^ and Armelle Penhoat^1*^

^1^CarMeN Laboratory, Inserm U1060, INRAE U1397, Univ-Lyon, Université Claude Bernard Lyon-1, Pierre Bénite, France, ^2^Service de Biochimie et de Biologie Moléculaire, Centre de Biologie et de Pathologie Est, Hospices Civils de Lyon, Bron, France

| Gene name | Forward (5’-3’) | Reverse (3’-5’) | Gene ID |
| --- | --- | --- | --- |
| TBP | AGACCATTGCACTTCGTGCC | CCTGTGCACACCATTTTCCC | 6908 |
| CERS2 | TCTTCAGCATTGCCTCTGAT | CCGCGTAGTTAAACATCTTG | 29956 |
| CERS4 | GATGGACTCGTAGTATGTGG | TGTTACACGATTCCTCTGAC | 79603 |
| CERS5 | GGCCTTCTATTGGTCCCTTA | TTCCCACTCGAACCATATTG | 91012 |
| CERS6 | CCTGTTTGTTATGTTTGCCG | AGACCAGAAGCAGTTCAACC | 253782 |
| SGMS1 | CCTGGTATGCATTTCAACTG | TGGCCGCTGTACAGATAGTC | 259230 |
| SGMS2 | CAATAGTGGGACGCAGATTC | GGACAATCCACCACCAGAAA | 166929 |

Supplemental Table S1. Primer sequences used in RT-qPCR experiments.

A-Ceramides Sphingomyelins

| **CER**  ***Proposed Structure*** | MW | MRM (m/z)  [M+H-H_2_O]+ |
| --- | --- | --- |
|  |  |  |
| CER (d18:1/16:1) | 535.5 | 518.5>264.5 |
| CER (d18:1/16:0) | 537.5 | 520.5>264.5 |
| CER (d18:1/18:1) | 563.5 | 546.5>264.5 |
| CER (d18:1/18:0) | 565.5 | 548.5>264.5 |
| CER (d18:1/19:0) | 579.5 | 562.5>264.5 |
| CER (d18:1/20:1) | 591.5 | 574.5>264.5 |
| CER (d18:1/20:0) | 593.5 | 576.5>264.5 |
| CER (d18:1/21:0) | 607.5 | 590.5>264.5 |
| CER (d18:1/22:1) | 619.5 | 602.5>264.5 |
| CER (d18:1/22:0) | 621.5 | 604.5>264.5 |
| CER (d18:1/23:0) | 635.5 | 618.5>264.5 |
| CER (d18:1/24:1) | 647.5 | 630.5>264.5 |
| CER (d18:1/24:0) | 649.5 | 632.5>264.5 |
| CER (d18:1/25:0) | 663.5 | 646.5>264.5 |
| CER (d18:1/26:1) | 675.5 | 658.5>264.5 |
| CER (d18:1/26:0) | 677.5 | 660.5>264.5 |

| **SM**  ***Proposed Structure*** | MW | MRM (m/z)  [M+H]+ |
| --- | --- | --- |
|  |  |  |
| SM (d18:1/16:1) | 700.8 | 701.8>184.0 |
| SM (d18:1/16:0) | 702.8 | 703.8>184.0 |
| SM (d18:1/18:1) | 728.8 | 729.8>184.0 |
| SM (d18:1/18:0) | 730.8 | 731.8>184.0 |
| SM (d18:1/19:0) | 744.8 | 745.8>184.0 |
| SM (d18:1/20:1) | 756.8 | 757.8>184.0 |
| SM (d18:1/20:0) | 758.8 | 759.8>184.0 |
| SM (d18:1/21:0) | 772.8 | 773.8>184.0 |
| SM (d18:1/22:1) | 784.8 | 785.8>184.0 |
| SM (d18:1/22:0) | 786.8 | 787.8>184.0 |
| SM (d18:1/23:0) | 800.8 | 801.8>184.0 |
| SM (d18:1/24:1) | 812.8 | 813.8>184.0 |
| SM (d18:1/24:0) | 814.8 | 815.8>184.0 |
| SM (d18:1/25:0) | 828.8 | 829.8>184.0 |
| SM (d18:1/26:1) | 840.8 | 841.8>184.0 |
| SM (d18:1/26:0) | 842.8 | 843.8>184.0 |

| **CER-d9**  ***Proposed Structure*** | MW | MRM (m/z)  [M+H-H_2_O]+ |
| --- | --- | --- |
|  |  |  |
| CER-d9 (d18:1/16:1) | 544.5 | 527.5>273.5 |
| CER-d9 (d18:1/16:0) | 546.5 | 529.5>273.5 |
| CER-d9 (d18:1/18:1) | 572.5 | 555.5>273.5 |
| CER-d9 (d18:1/18:0) | 574.5 | 557.5>273.5 |
| CER-d9 (d18:1/19:0) | 588.5 | 571.5>273.5 |
| CER-d9 (d18:1/20:1) | 600.5 | 583.5>273.5 |
| CER-d9 (d18:1/20:0) | 602.5 | 585.5>273.5 |
| CER-d9 (d18:1/21:0) | 616.5 | 599.5>273.5 |
| CER-d9 (d18:1/22:1) | 628.5 | 611.5>273.5 |
| CER-d9 (d18:1/22:0) | 630.5 | 613.5>273.5 |
| CER-d9 (d18:1/23:0) | 644.5 | 627.5>273.5 |
| CER-d9 (d18:1/24:1) | 656.5 | 639.5>273.5 |
| CER-d9 (d18:1/24:0) | 658.5 | 641.5>273.5 |
| CER-d9 (d18:1/25:0) | 672.5 | 655.5>273.5 |
| CER-d9 (d18:1/26:1) | 684.5 | 667.5>273.5 |
| CER-d9 (d18:1/26:0) | 686.5 | 669.5>273.5 |

B- Ceramides-d9 Sphingomyelins-d9

| **SM-d9**  ***Proposed Structure*** | MW | MRM (m/z)  [M+H]+ |
| --- | --- | --- |
|  |  |  |
| SM-d9 (d18:1/16:1) | 709.8 | 710.8>184.0 |
| SM-d9 (d18:1/16:0) | 711.8 | 712.8>184.0 |
| SM-d9 (d18:1/18:1) | 737.8 | 738.8>184.0 |
| SM-d9 (d18:1/18:0) | 739.8 | 740.8>184.0 |
| SM-d9 (d18:1/19:0) | 753.8 | 754.8>184.0 |
| SM-d9 (d18:1/20:1) | 765.8 | 766.8>184.0 |
| SM-d9 (d18:1/20:0) | 767.8 | 768.8>184.0 |
| SM-d9 (d18:1/21:0) | 781.8 | 782.8>184.0 |
| SM-d9 (d18:1/22:1) | 793.8 | 794.8>184.0 |
| SM-d9 (d18:1/22:0) | 795.8 | 796.8>184.0 |
| SM-d9 (d18:1/23:0) | 809.8 | 810.8>184.0 |
| SM-d9 (d18:1/24:1) | 821.8 | 822.8>184.0 |
| SM-d9 (d18:1/24:0) | 823.8 | 824.8>184.0 |
| SM-d9 (d18:1/25:0) | 837.8 | 838.8>184.0 |
| SM-d9 (d18:1/26:1) | 849.8 | 850.8>184.0 |
| SM-d9 (d18:1/26:0) | 851.8 | 852.8>184.0 |

Supplemental Table S2. Transitions used for quantification of canonic CER and SM species (A) and deuterated CER and SM species (B) by tandem mass spectrometry.

Supplemental Fig S1. Kinetics of basolateral TG secretion in the basolateral medium of Caco-2/TC7 cells.

Caco-2/TC7 cells were incubated with mixed micelles during different times. Data represent the mean ± SEM of one experiment realized in triplicate. Each time represents three independent wells. Values bearing different superscript letters are significantly different.

Supplemental Fig S2. Molecular species concentrations of unlabeled Cer and unlabeled SM in cells.

Unlabeled Cer (A) and unlabeled SM (B) molecular species concentrations in Caco-2/TC7 cells incubated for 16h with mixed micelles (MM) enriched or not with SPH-d9 (MM+SPH-d9) or with SPH-d9 and C23:0 (MM+SPH-d9+C23:0). Cer molecular species annotations were based on the assumption that sphingosine d18:1 is the major sphingoid base. Data expressed as nmol/g protein, represent the mean ± SEM of one experiment realized in quadruplicate.

Asterisks represent a significant difference compared to MM-enriched cells (*P<0.05).
